# Beyond one-size-fits-all evidence: integrating person-centered analyses into clinical research

**DOI:** 10.3389/fpubh.2026.1961483

**Published:** 2026-10-14

**Authors:** Federica Bonazza, Angela Sorgente, Lidia Borghi, Elena Vegni, Sara Molgora

**Affiliations:** 1 Department of Psychology, Catholic University of the Sacred Heart, Milano, Italy; 2 Department of Health Sciences, University of Milan, Milan, Italy

**Keywords:** clinical research, patient-centered care, person-centered analysis, precision medicine, randomized controlled trial

## Introduction

Two theoretical frameworks have been particularly influential in shaping medicine: the disease-centered and patient-centered models. The disease-centered, or biomedical, model conceptualizes disease as a deviation from the established biological norm and prioritizes diagnostic accuracy and therapeutic efficacy (1). Within this paradigm, the primary aim of medical practice is to identify pathological processes as precisely as possible and apply evidence-based interventions that target the disease itself. However, over decades, accumulated evidence has revealed the limitations of this approach, particularly when dealing with chronic conditions and patients whose needs cannot be fully captured by biological indicators alone (2). A key contribution in addressing these limitations was the biopsychosocial model (3), which argued that illness cannot be understood and managed without considering its psychological and social dimensions. This perspective has prompted the progressive integration of the patient-centered model (4), which does not replace the traditional paradigm but expands it by adding a broader understanding of care.

Rather than focusing exclusively on the disease, the patient-centered model promotes a form of care that considers both the biological dimension of illness and the subjective illness experience. In this view, patients are addressed holistically: their clinical condition remains central, but their ideas, preferences, values, expectations, and needs are also taken into account (2). This shift recognizes that individuals with the same diagnosis may experience their condition differently and may therefore require different forms of support and intervention. For instance, although patients share a diagnosis of diabetes, they often manifest differences in illness perception, health literacy, self-management, emotional burden, and engagement with care (5, 6). Consequently, two patients with the same clinical profile may differ substantially in their understanding of their disease, response to treatment, and daily disease management. Throughout this commentary, we will continue using diabetes as an illustrative example, because keeping the disease constant allows us to show how the same condition can be studied through different analytic approaches. However, these arguments can be applied to clinical research more broadly.

## Variable-centered and person-centered approaches

This conceptual distinction has important methodological implications. The scientific literature distinguishes between variable-centered and person-centered analytic approaches, which address different but related questions. Variable-centered statistical analyses assume that participants are drawn from a “*single population for which a single set of averaged parameters can be estimated*” (7). For example, when we calculate the correlation between two variables, we obtain a single parameter (e.g., r = 0.43) that summarizes how strongly the variables are associated, on average, in the population of interest, despite some degree of heterogeneity across individuals. However, this does not mean that variable-centered analyses are blind to heterogeneity. Specific techniques within this approach can be used to examine differences across participants. For example, we can test whether the effect of a treatment depends on participants' baseline characteristics through treatment-by-moderator interactions, whereas multilevel models with random slopes allow effects to vary across individuals or clusters (8, 9).

The person-centered approach offers a complementary perspective to investigate heterogeneity. It includes statistical techniques that assume that a study sample may include multiple subgroups “*characterized by different sets of parameters*” (7). Among these, latent class and latent profile analyses make it possible to identify distinct subgroups within the same sample, defined by different patterns across the same set of variables. Thus, heterogeneity is the central focus of these analyses: rather than assuming that a single set of average parameters adequately characterizes the entire population, they seek to divide it into relatively homogeneous subgroups that differ from one another in how the selected variables combine to form distinct response patterns. Rather than examining characteristics in isolation, these methods summarize how they co-occur among individuals. The variables used to distinguish between groups may include sociodemographic characteristics and/or patient-reported measures that are clinically relevant to the patient-centered model. For instance, Qu and colleagues (10) examined a cohort of patients with diabetes and identified patient profiles based on attitudes toward the disease, including trust in physicians, patient activation, social support, distress, and perceived discrimination. Their latent class analysis identified three groups. The first group included “highly engaged patients”, who reported strong engagement and good self-efficacy in doctor–patient interactions. The second group described “moderately engaged patients,” who showed lower levels of trust and engagement, but higher levels of distress and perceived discrimination. The third group comprised “depressed patients,” who experienced the highest levels of distress and perceived discrimination, as well as the highest rate of depressive symptoms.

Once identified, latent subgroups can be eventually entered as moderators in variable-centered models, making it possible to test formally whether treatment effects differ across patient profiles (11). Thus, the variable-centered and person-centered approaches can be combined.

## Discussion

The person-centered approach is closely aligned with the principles of patient-centered care, sharing the core assumption that patient populations are heterogeneous, even when individuals share the same diagnosis. This convergence is conceptually important because it suggests that clinical care and research should both recognize that the same disease may be experienced, managed, and responded to in different ways. Despite this methodological and conceptual affinity, medical research remains largely anchored to the variable-centered approach. A clear example is the way in which randomized controlled trials (RCTs) are typically analyzed. Although RCTs remain the gold standard for evaluating the effectiveness of interventions, conventional RCT analyses generally focus on estimating the average intervention effect across the study population. This approach is useful for estimating the overall effectiveness of an intervention. The average treatment effect, however, is by definition a population-level estimate. This does not imply that every participant benefits to the same extent, nor it indicate whether the benefit is widespread or concentrated amongst certain patients. For instance, the RCT by Cheng and colleagues (12) demonstrated the value of an empowerment-based self-management intervention for patients with diabetes and reported a moderate and significant improvement in empowerment and quality of life compared with a control group receiving only standard care. In line with standard practice for RCTs, these results were reported as mean effects across the entire study population. Whilst appropriate for establishing efficacy, this estimate leaves open the question of whether patients with different baseline characteristics – for example, in terms of diabetes-related distress, health literacy, or engagement with care – benefited to a similar extent.

The scientific literature is increasingly attentive to heterogeneity of treatment effects, that is, the non-random variation in the magnitude or direction of a treatment effect across patients (13). This attention is reflected in the growth of personalized and precision medicine, which aims to match treatments to patients' individual characteristics rather than to the average patient (13), and in the development of dedicated methods to assess how treatment effects vary across patients within trials (14, 15). Thus, latent class and latent profile analyses are well suited to this purpose.

In recent years, an increasing number of studies have already taken steps in this direction. For example, Qu and colleagues (10) tested a self-management intervention across three identified subgroups. Improvements in distress and quality of life were observed only among the “depressed patients” group, whereas the “highly-engaged patients” and “moderately-engaged patients” groups showed limited changes, mainly in body mass index. These findings suggest that the same intervention may have different effects depending on the psychological, behavioral, and social characteristics of the patients to whom it is delivered. Although we focus mainly on diabetes as an illustrative example, similar findings were found for other diseases. For instance, in a study involving a sample of patients with acute respiratory distress syndrome, latent class analysis identified two subphenotype that responded differently to ventilation strategy, fluid management, and simvastatin (16, 17).

Patient-centered care is widely regarded as a cornerstone of high-quality medical practice; however, its principles remain insufficiently integrated into clinical research. Integrating person-centered methodology into RCTs would help bridge this gap and create a more direct dialogue between clinical practice and research. This approach could generate more nuanced insights into which psychosocial—and even medical—interventions are effective for which patients, thereby supporting the design of more tailored and clinically meaningful interventions. Since tailored care is widely promoted, person-centered methodology may provide important tools to guide its development and move beyond one-size-fits-all interventions.

Taken together, these considerations have direct implications for public health, particularly for interventions designed and evaluated at the population level. The populations targeted by such interventions are, in fact, rarely homogeneous. Differences in health literacy, illness perceptions, social support, self-management skills, and psychosocial burden can influence both engagement with an intervention and its effectiveness. Recognizing this heterogeneity may therefore be important for developing interventions that are not only effective on average, but also feasible and equitable across different patient groups. From this perspective, person-centered analyses hold considerable promise for clinical practice and for the evidence-based development of patient-centered interventions.

Before drawing this conclusion, however, it is important to acknowledge the methodological challenges associated with person-centered analyses. Despite their potential, latent class and latent profile analyses have several limitations that should be considered when they are applied in clinical research. Firstly, they require relatively large samples. The accuracy of class identification depends on the sample size, the number and quality of indicators, and the degree of separation between classes; small classes may not be detected or may be estimated inaccurately (18). Secondly, the choice of the number of classes may be partly subjective. Researchers rely on a combination of statistical fit indices; however, these criteria do not always agree (19). Thirdly, class solutions may not be replicable across samples, particularly when sample are not representative (20); therefore, profiles identified in one study should be validated on independent data before being used to guide clinical care. Fourthly, assigning participants to the most likely class ignores the uncertainty of the classification, which may introduce bias into subsequent analyses unless appropriate methods are used (21). Overall, latent profiles cannot be identified as fixed labels that replace attention to the individual patient, they are best understood as useful summaries of heterogeneity that can inform research and care.

Despite these methodological challenges, we argue that person-centered analyses provide a plausible and promising avenue for improving clinical practice and the development of tailored interventions. Existing initiatives already illustrate the practical value of identifying groups with different configurations of strengths and needs to guide intervention design. The Ophelia (Optimizing Health Literacy and Access) process, for instance, uses multidimensional health literacy data to identify groups of people with distinct strengths and needs, which then guide the co-design of tailored health literacy actions (22, 23). Latent class and latent profile analyses could further strengthen profile-based approaches of this kind by providing model-based criteria for selecting the number of profiles, estimating individuals' probabilities of profile membership, and examining whether the identified profiles replicate across populations.
